# Multiple Scale Homogenisation of Nutrient Movement and Crop Growth in Partially Saturated Soil

**DOI:** 10.1007/s11538-019-00656-3

**Published:** 2019-08-22

**Authors:** Simon J. Duncan, Keith R. Daly, Daniel M. McKay Fletcher, Siul Ruiz, Paul Sweeney, Tiina Roose

**Affiliations:** 1 School of Engineering, Faculty of Engineering and Physical Sciences, University of Southampton, Southampton, SO17 1BJ UK; 2 Syngenta, Jealott’s Hill, Bracknell, RG42 6EY UK

**Keywords:** Homogenisation, Deforming geometry, Diffusion, Solute movement

## Abstract

In this paper, we use multiple scale homogenisation to derive a set of averaged macroscale equations that describe the movement of nutrients in partially saturated soil that contains growing potato tubers. The soil is modelled as a poroelastic material, which is deformed by the growth of the tubers, where the growth of each tuber is dependent on the uptake of nutrients via a sink term within the soil representing root nutrient uptake. Special attention is paid to the reduction in void space, resulting change in local water content and the impact on nutrient diffusion within the soil as the tubers increase in size. To validate the multiple scale homogenisation procedure, we compare the system of homogenised equations to the original set of equations and find that the solutions between the two models differ by $\lesssim 2 \%$. However, we find that the computation time between the two sets of equations differs by several orders of magnitude. This is due to the combined effects of the complex three-dimensional geometry and the implementation of a moving boundary condition to capture tuber growth.

## Introduction

Application of solutes such as fertilisers and pesticides is important in modern agricultural practices (Godfray et al. 2010). However, more efficient solute application is needed in order to mitigate growing costs of fertilisers and environmental pollution, *i.e.* fertiliser and pesticide buffering, leaching and run off (Godfray et al. 2010). Hence, understanding water and solute movement in soil is vital for determining sustainable crop production for long-term food security (Comas et al. 2013). To aid with this goal, mathematical modelling of soil systems has been studied increasingly in recent years (Vereecken et al. 2016), since this offers one method to investigate plant–soil interactions while reducing time and resources compared to standard experimental practices. Combining mathematical modelling with traditional experiments allows us to efficiently improve our understanding of plant–soil interactions (Roose et al. 2016; Daly et al. 2017). This can lead to further improvement of agricultural techniques for greater crop yield while minimising waste of resources.

Mathematical modelling of soil systems covers a wide range of spatial scales, including pore, plant and field scales (Darrah et al. 2006; Hopmans et al. 2002). As such, when studying transport of water and solutes in soil, complex geometries are often required to capture the intrinsic details contained in the microscopic structure of the scale that is considered. This typically requires vast amounts of computation time and resources (Daly and Roose 2018). Hence, it is often favourable to construct an averaged macroscopic geometry so that the macroscale transport properties can be attained directly from the microscale information (Bruna and Chapman 2015). One technique that is frequently used to obtain macroscale movement of fluids and solutes in soil or other porous media is multiple scale homogenisation (Hornung 2012). This mathematical technique is a method of devising a system of averaged macroscopic equations that are parameterised by associated cell problems, which are derived from the inherent microscopic structure of the domain (Pavliotis and Stuart 2008).

Multiple scale homogenisation has been successfully used in a wide range of porous media and soil applications, including modelling saturated fluid flow (Keller 1980), two-phase fluid flow (Daly and Roose 2015), wave propagation in poroelastic materials (Sharma 2007) and single-phase fluid flow in double porosity systems (Arbogast et al. 1990). One application that has been increasingly studied in recent years is homogenisation of moving interfaces for first- and second-order partial differential equations (Cardaliaguet et al. 2009; Lions and Souganidis 2005). Although there has been extensive research on the mathematical theory for the homogenisation of moving interfaces, few applications have been explored.

In this study, we demonstrate the utility of homogenisation by modelling the growth of potato tubers in soil, in which the growth is dependent on the quantity of nutrients the plant is able to draw up from the soil. We model the soil as a poroelastic material, such that any growth from a single crop will influence the water content adjacent to the plant and therefore the movement of nutrients in the vicinity. We use a combination of poroelastic theory and the diffusion equation in porous media to model the movement of nutrients in a deforming soil environment. We develop a series of approximate equations to describe nutrient movement, growth in tuber size and global nutrient uptake in soil.

There has been previous research which studied the effect of diffusion with spatially varying objects in porous media (Bruna and Chapman 2015), in which Rayleigh’s multipole method was used to determine a spatially dependent effective diffusion coefficient based on the size of the sphere within the microscopic periodic geometry (Rayleigh 1892). Here, we extend this idea to model both spatially and temporally varying objects in poroelastic media, which are coupled to the diffusion of the species within the material itself.

For simplicity, we choose to model the tubers as spherical objects in soil; however, this can be extended to any 3D geometry, including, but not limited to, ovoids, capsules and cylinders. To validate the homogenisation procedure, we compare the solution of the homogenised equations against the full system for a series of case studies. This shows the homogenised equations successfully capture the growth of each tuber and the change in nutrient diffusion from the reduction of volume within the domain.

## Theory

### Three-Phase Poroelastic Soils

**Fig. 1:**
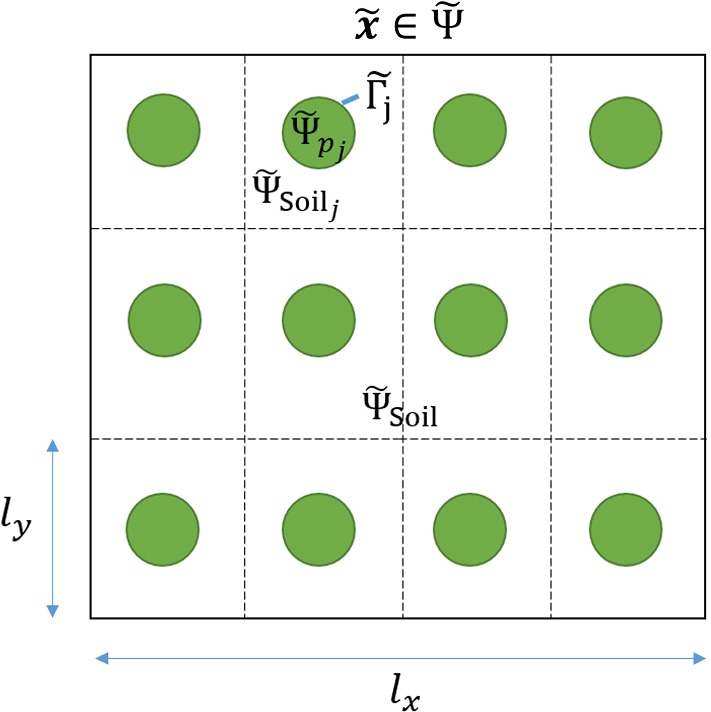
Schematic of a dimensional poroelastic domain, where ${\tilde{{\varvec{\Psi }}}}$ is the total domain, ${\tilde{{\varvec{\Psi }}}}_{\text {Soil}}$ is the deformable poroelastic soil domain, ${\tilde{{\varvec{\Psi }}}}_{p_j}$ are the potato tubers, ${\tilde{{\varvec{\Psi }}}}_{\text {Soil}_j}$ are the poroelastic soil subdomains adjacent to each tuber and ${\tilde{{\varvec{\Gamma }}}}_j$ are the boundaries between ${\tilde{{\varvec{\Psi }}}}_{p_j}$ and ${\tilde{{\varvec{\Psi }}}}_{\text {Soil}}$. In addition, $l_x$ is the macroscale and $l_y$ is the microscale (Color figure online)

Let ${\tilde{{\varvec{\Psi }}}} \subset {\mathbb {R}}^3$ be an open bounded subset representing a soil system (Fig. 1) that contains *N* potato tubers. We define ${\tilde{{\varvec{\Psi }}}}= {\tilde{{\varvec{\Psi }}}}_{\text {Soil}} \cup \sum _{j=1}^N {\tilde{{\varvec{\Psi }}}}_{p_j}$, where ${\tilde{{\varvec{\Psi }}}}_{\text {Soil}}$ is the deformable poroelastic soil domain that is composed of water, air and solid components, and ${\tilde{{\varvec{\Psi }}}}_{p_j}$ are the $j=1,\dots ,N$ potato tubers each with a boundary ${\tilde{{\varvec{\Gamma }}}}_j$.

To describe the deformable poroelastic soil domain ${\tilde{{\varvec{\Psi }}}}_{\text {Soil}}$, we impose a system of equations that describe a three-phase poroelastic domain. To derive the system of equations, we use the conservation laws for mass and momentum. The conservation of mass equations for the three phases of air, water and soil solid is

1 $$\begin{aligned}&\partial _{{\tilde{t}}}\phi _a = - \tilde{\varvec{\nabla }} \cdot \left( \phi _a {\tilde{\mathbf {v}}}_a \right) , \quad {\tilde{\mathbf {x}}} \in {\tilde{{\varvec{\Psi }}}}_{\text {Soil}} , \end{aligned}$$

2 $$\begin{aligned}&\partial _{{\tilde{t}}}\phi _w = - \tilde{\varvec{\nabla }} \cdot \left( \phi _w {\tilde{\mathbf {v}}}_w \right) - \lambda _c({\tilde{p}}_w-p_r), \quad {\tilde{\mathbf {x}}} \in {\tilde{{\varvec{\Psi }}}}_{\text {Soil}} , \end{aligned}$$

3 $$\begin{aligned}&\partial _{{\tilde{t}}} \phi _s = -\tilde{\varvec{\nabla }} \cdot \left( \phi _s {\tilde{\mathbf {v}}}_s \right) , \quad {\tilde{\mathbf {x}}} \in {\tilde{{\varvec{\Psi }}}}_{\text {Soil}} , \end{aligned}$$

4 $$\begin{aligned}&\phi _a + \phi _w + \phi _s = 1, \end{aligned}$$

where $\phi _a$ is the volumetric air content, $\phi _w$ is the volumetric water content, $\phi _s$ is the volumetric soil solid content, ${\tilde{\mathbf {v}}}_a$ is the air velocity, ${\tilde{\mathbf {v}}}_w$ is the water velocity, ${\tilde{\mathbf {v}}}_s$ is the velocity of the soil solid component and ${\tilde{p}}_w$ is the soil water pore pressure. Water uptake in our simulations is assumed to be dominated by transport through symplastic pathways, thus passively taken up by pressure gradients in the root xylem (Roose and Fowler 2004b). The ratio between the cortex and the xylem hydraulic conductivities along with the root surface area density is characterised by $\lambda _c$, and the root xylem pressure is expressed as $p_r$. Roots are assumed to be uniformly distributed throughout the soil domain. We note that we neglect the impact that tuber growth has on the root system. The expression $-\, \lambda _c({\tilde{p}}_w-p_r)$ represents water uptake by plant roots.

Furthermore, Darcy’s law for the relative phase velocity of air and water is written as

5 $$\begin{aligned} \phi _a \left( {\tilde{\mathbf {v}}}_a - {\tilde{\mathbf {v}}}_s \right)= & {} - \frac{\kappa _a}{\mu _a} \tilde{\varvec{\nabla }} {\tilde{p}}_a, \quad {\tilde{\mathbf {x}}} \in {\tilde{{\varvec{\Psi }}}}_{\text {Soil}}, \end{aligned}$$

6 $$\begin{aligned} \phi _w \left( {\tilde{\mathbf {v}}}_w - {\tilde{\mathbf {v}}}_s \right)= & {} - \frac{\kappa _w}{\mu _w} \tilde{\varvec{\nabla }} {\tilde{p}}_w, \quad {\tilde{\mathbf {x}}} \in {\tilde{{\varvec{\Psi }}}}_{\text {Soil}}, \end{aligned}$$

where ${\tilde{p}}_a$ is the soil air pore pressure, $\kappa _a$ and $\kappa _w$ are the air and water permeabilities, respectively, and $\mu _a$ and $\mu _w$ are the viscosities of air and water, respectively.

The air and water pressures ${\tilde{p}}_a$ and ${\tilde{p}}_w$, and the air and water volume fractions $\phi _a$ and $\phi _w$ are related via the van Genuchten saturation expression (Van Genuchten 1980)

7 $$\begin{aligned} S_w =\Bigg [ \left( \frac{{\tilde{p}}_a-{\tilde{p}}_w}{p_c} \right) ^{\frac{1}{1-m}} +1 \Bigg ]^{-m}, \end{aligned}$$

where $$S_w = \phi _w/(\phi _w+\phi _a) $$ is the relative water saturation, $$p_c $$ is the characteristic suction pressure and *m* is the van Genuchten parameter.

The conservation of momentum equation is (Wang 2000)

8 $$\begin{aligned} \tilde{\varvec{\nabla }} \cdot {\mathfrak {G}}= & {} 0 , \quad {\tilde{\mathbf {x}}} \in {\tilde{{\varvec{\Psi }}}}_{\text {Soil}}, \end{aligned}$$

9 $$\begin{aligned} {\mathfrak {G}}= & {} G \bigg [ \left( \tilde{\varvec{\nabla }} {\tilde{\mathbf {u}}}_s \right) + \left( \tilde{\varvec{\nabla }} {\tilde{\mathbf {u}}}_s \right) ^T + \frac{\nu }{1-2 \nu } \tilde{\varvec{\nabla }} \cdot {\tilde{\mathbf {u}}}_s {\mathfrak {T}} \bigg ] - S_w {\tilde{p}}_w {\mathfrak {T}} - S_a {\tilde{p}}_a {\mathfrak {T}} , \end{aligned}$$

where ${\mathfrak {G}}$ is the stress tensor, ${\tilde{\mathbf {u}}}_s$ is the displacement of the solid soil matrix, *G* is the shear modulus of the soil solid, $\nu$ is the Poisson ratio, $S_a=\phi _a/(\phi _w+\phi _a)$ is the relative air saturation and ${\mathfrak {T}}$ is the identity tensor. The displacement ${\tilde{\mathbf {u}}}_s$ is related to ${\tilde{\mathbf {v}}}_s$ by the relationship

10 $$\begin{aligned} {\tilde{\mathbf {v}}}_s= & {} \partial _{{\tilde{t}}} {\tilde{\mathbf {u}}}_s. \end{aligned}$$

The system of Eqs. (1)–(10) completes a full mathematical description of a three-phase poroelastic soil.

### Diffusion of Nutrients in Soil

Solutes such as nutrients typically exist in one of two states in soil, either sorbed to the soil solid surfaces or dissolved in the pore water (Roose et al. 2001). We state that the nutrient concentration in the sorbed state follows a reversible linear binding reaction such that,

11 $$\begin{aligned} \partial _{{\tilde{t}}} {\tilde{c}}_s= & {} d_s, \quad {\tilde{\mathbf {x}}} \in {\tilde{{\varvec{\Psi }}}}_{\text {Soil}} , \end{aligned}$$

where ${\tilde{c}}_s$ is the sorbed nutrient concentration and $d_s$ is the net transfer rate to the sorbed phase from the pore water phase. From the conservation of mass, the rate of change of the nutrient concentration in the pore water phase is

12 $$\begin{aligned} \partial _{{\tilde{t}}} (\phi _w {\tilde{c}})= & {} \tilde{\varvec{\nabla }} \cdot \left( D \phi _w \tilde{\varvec{\nabla }} {\tilde{c}} \right) + d_l - g{\tilde{c}}, \quad {\tilde{\mathbf {x}}} \in {\tilde{{\varvec{\Psi }}}}_{\text {Soil}} , \end{aligned}$$

where $${\tilde{c}}$$ is the nutrient concentration in pore water, $$d_l $$ is the net transfer rate to the pore water phase from the sorbed phase, *D* is the diffusion coefficient and *g* is the nutrient uptake rate by plant roots. Adding (11) and (12) yields

13 $$\begin{aligned} \partial _{{\tilde{t}}} ({\tilde{c}}_s + \phi _w {\tilde{c}})= & {} \tilde{\varvec{\nabla }} \cdot \left( D \phi _w\tilde{\varvec{\nabla }} {\tilde{c}} \right) + d_s + d_l - g{\tilde{c}}, \quad {\tilde{\mathbf {x}}} \in {\tilde{{\varvec{\Psi }}}}_{\text {Soil}}. \end{aligned}$$

We assume there is a direct jump between the nutrients in the two states with no intermediate phase, such that $d_s + d_l = 0$. Furthermore, we define $d_s$

14 $$\begin{aligned} d_s = k_a {\tilde{c}} - k_d {\tilde{c}}_s = \partial _{{\tilde{t}}} {\tilde{c}}_s, \end{aligned}$$

where $$k_a $$ is the adsorption rate of the nutrient in solution and $$k_d $$ is the desorption rate. We assume $$k_d$$ is sufficiently large such that $$d_s/k_d = \partial _{{\tilde{t}}} {\tilde{c}}_s/k_d \ll 1$$ and $$k_a \sim k_d$$, then

15 $$\begin{aligned} {\tilde{c}}_s = b {\tilde{c}}, \end{aligned}$$

where $b=k_a/k_d$ is the buffer power of the nutrient (Nye and Tinker 1977; Barber 1995; Roose and Fowler 2004a). This leads to the governing equation for nutrient movement in terms of ${\tilde{c}}$ only, *i.e.*

16 $$\begin{aligned} (\phi _w + b)\partial _{{\tilde{t}}} {\tilde{c}} + {\tilde{c}}\partial _{{\tilde{t}}} \phi _w = \tilde{\varvec{\nabla }} \cdot \left( D \phi _w \tilde{\varvec{\nabla }} {\tilde{c}} \right) - g{\tilde{c}}, \quad {\tilde{\mathbf {x}}} \in {\tilde{{\varvec{\Psi }}}}_{\text {Soil}} . \end{aligned}$$

### Boundary Conditions

Here, we define a series of boundary conditions on the interfaces ${\tilde{{\varvec{\Gamma }}}}_j$, *i.e.* between the deformable poroelastic soil domain ${\tilde{{\varvec{\Psi }}}}_{\text {Soil}}$ and the potato tubers ${\tilde{{\varvec{\Psi }}}}_{p_j}$. To describe nutrient interaction on ${\tilde{{\varvec{\Gamma }}}}_j$, we impose a zero flux condition, as the potato tubers take up nutrients through their rooting systems and not through the tuber surfaces:

17 $$\begin{aligned} {\hat{\mathbf {n}}} \cdot \left( D \phi _w \tilde{\varvec{\nabla }} {\tilde{c}} \right) = 0, \quad {\tilde{\mathbf {x}}} \in {\tilde{{\varvec{\Gamma }}}}_j , \end{aligned}$$

where ${\hat{\mathbf {n}}}$ is the unit normal vector pointing out of the geometry. Furthermore, on ${\tilde{{\varvec{\Gamma }}}}_j$ we assume the soil solid is displaced normally to the direction of the growing tuber, hence

18 $$\begin{aligned} \left( 2 {\hat{\mathbf {n}}} \otimes {\hat{\mathbf {n}}} - {\mathfrak {T}} \right) \cdot {\tilde{\mathbf {u}}}_s = {\hat{\mathbf {n}}} \xi _j, \quad {\tilde{\mathbf {x}}} \in {\tilde{{\varvec{\Gamma }}}}_j , \end{aligned}$$

where $\xi _j$ is the displacement of the $j^{\text {th}}$ tuber given by,

19 $$\begin{aligned} \xi _j = {\tilde{r}}_j - r^*, \end{aligned}$$

where $$ r^*$$ is the initial radius of the tubers and $${\tilde{r}}_j $$ is the radius of the *j*th tuber, which is related to the total amount of nutrients taken up by the roots. The growth of each tuber is expressed as

20 $$\begin{aligned} \partial _{{\tilde{t}}} {\tilde{V}}_j = \alpha \int _{{\tilde{{\varvec{\Psi }}}}_{\text {Soil}_j} } g {\tilde{c}} \ d {\tilde{{\varvec{\Psi }}}}_{\text {Soil}_j} , \end{aligned}$$

where ${\tilde{V}}_j$ is the tuber volume, $\alpha$ is the ratio between the rate of growth and uptake and ${\tilde{{\varvec{\Psi }}}}_{\text {Soil}_j}$ is the volume of soil adjacent to each potato tuber *j* (see Fig. 1). Here, we model the early-stage development of potato tubers (diameter 5–7 cm); hence, we approximate the tubers shape to be spherical. Therefore, Eq. (20) can be written in terms of the radius ${\tilde{r}}_j$ only, *i.e.*

21 $$\begin{aligned} \partial _{{\tilde{t}}} {\tilde{r}}_j = \frac{\alpha }{4 \pi {\tilde{r}}_j^2} \int _{{\tilde{{\varvec{\Psi }}}}_{\text {Soil}_j} } g {\tilde{c}} \ d {\tilde{{\varvec{\Psi }}}}_{\text {Soil}_j} . \end{aligned}$$

We state the water and air components of ${\tilde{{\varvec{\Psi }}}}_{\text {Soil}}$ do not penetrate the tubers ${\tilde{{\varvec{\Psi }}}}_{p_{j}}$; thus, we require the Darcy velocities normal to the interface to be zero, *i.e.*

22 $$\begin{aligned} {\hat{\mathbf {n}}} \cdot \left( \frac{\kappa _w}{\mu _w} \tilde{\varvec{\nabla }} {\tilde{p}}_w \right)= & {} 0, \quad {\tilde{\mathbf {x}}} \in {\tilde{{\varvec{\Gamma }}}}_j , \end{aligned}$$

23 $$\begin{aligned} {\hat{\mathbf {n}}} \cdot \left( \frac{\kappa _a}{\mu _a} \tilde{\varvec{\nabla }} {\tilde{p}}_a \right)= & {} 0, \quad {\tilde{\mathbf {x}}} \in {\tilde{{\varvec{\Gamma }}}}_j . \end{aligned}$$

Finally, on ${\tilde{{\varvec{\Gamma }}}}_j$ we assume the air and water velocities are equal to the growth of the tubers, hence

24 $$\begin{aligned} \left( 2 {\hat{\mathbf {n}}} \otimes {\hat{\mathbf {n}}} - {\mathfrak {T}} \right) \cdot {\tilde{\mathbf {v}}}_w= & {} {\hat{\mathbf {n}}} \partial _{{\tilde{t}}} {\tilde{r}}_j, \quad {\tilde{\mathbf {x}}} \in {\tilde{{\varvec{\Gamma }}}}_j , \end{aligned}$$

25 $$\begin{aligned} \left( 2 {\hat{\mathbf {n}}} \otimes {\hat{\mathbf {n}}} - {\mathfrak {T}} \right) \cdot {\tilde{\mathbf {v}}}_a= & {} {\hat{\mathbf {n}}} \partial _{{\tilde{t}}} {\tilde{r}}_j, \quad {\tilde{\mathbf {x}}} \in {\tilde{{\varvec{\Gamma }}}}_j . \end{aligned}$$

### Non-dimensionalisation

**Fig. 2:**
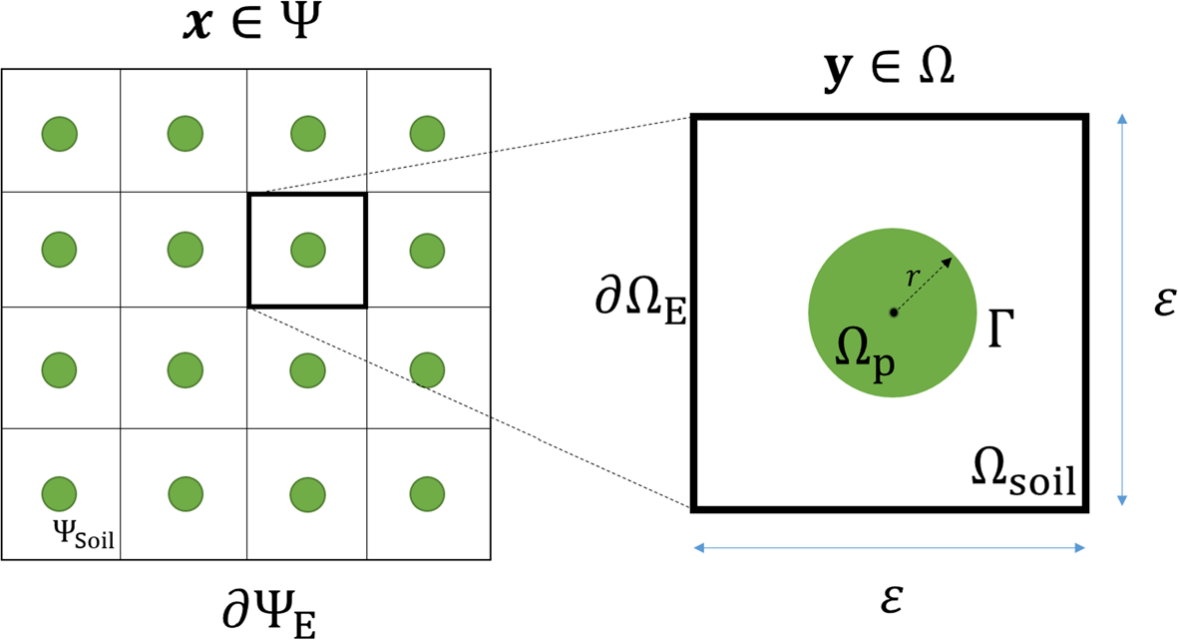
Schematic of the dimensionless macroscale domain $\mathbf {{{\varvec{\Psi }}}}$ and microscale domain ${{\varvec{\Omega }}}$, where $\mathbf {{{\varvec{\Psi }}}}_{\text {Soil}}$ is the poroelastic soil domain, $\partial {{\varvec{\Psi }}}_E$ is the external boundary of ${{\varvec{\Psi }}}$, ${{{\varvec{\Omega }}}}_{\text {Soil}}$ is the poroelastic domain composed of water, air and solid components, ${{\varvec{\Omega }}}_p$ is the potato tuber, ${{\varvec{\Gamma }}}$ is the boundary between ${{{\varvec{\Omega }}}}_{\text {Soil}}$ and ${{\varvec{\Omega }}}_p$, $\partial {{\varvec{\Omega }}}_E$ is the external boundary of the periodic cell and *r* is the radius of ${{\varvec{\Omega }}}_p$ (Color figure online)

To simplify the model and understand the magnitude of influence of each parameter, we non-dimensionalise the system of equations described above. We are interested in the macroscopic properties of the system of equations while retaining the influence of the microscopic structure. Hence, we identify two different length scales, the ‘microscopic’ length scale $l_y$ associated with the inner domain tuber geometry, and the macroscopic length scale $l_x$ associated with the full domain transport of water and nutrients. Under these scales, $l_y/l_x =\varepsilon \ll 1$. We choose to non-dimensionalise using the scaling

26 $$\begin{aligned} {\tilde{\mathbf {x}}}&= l_x {\mathbf {x}}, \ {\tilde{t}}=\frac{l_x^2}{D} t, \ {\tilde{\mathbf {u}}}_s = l_y {\mathbf {u}}_s, \ {\tilde{c}}=c_{\text {max}} c, \nonumber \\ {\tilde{p}}_i&=G p_i, \ {\tilde{\mathbf {v}}}_i = \frac{l_y D}{l_x^2} {\mathbf {v}}_i, \ {\tilde{r}}=l_y r, \end{aligned}$$

where $c_{\text {max}}$ is the maximum concentration of the nutrient applied to ${\tilde{{\varvec{\Psi }}}}_{\text {Soil}}$ and $i=\{w,a\}$. In (26) we use the macroscopic length scale $l_x$ as the spatial scaling to observe macroscale properties, the diffusion timescale $\frac{l_x^2}{D}$ for the time scaling and the shear modulus *G* for the pressure scaling. Shown in Fig. 2 are the non-dimensionalised macroscopic domain ${{\varvec{\Psi }}}$ and microscopic domain ${{\varvec{\Omega }}}$. It follows that the air, water and solid phase continuity equations become:

27 $$\begin{aligned} \partial _{t}\phi _a= & {} -\, \varepsilon {\varvec{\nabla }} \cdot \left( \phi _a {{\mathbf {v}}}_a \right) , \quad {{\mathbf {x}}} \in {{{\varvec{\Psi }}}}_{\text {Soil}} , \end{aligned}$$

28 $$\begin{aligned} \partial _{t}\phi _w= & {} -\, \varepsilon {\varvec{\nabla }} \cdot \left( \phi _w {{\mathbf {v}}}_w \right) - \overline{\lambda _c}({p}_w-\overline{p_r}) , \quad {{\mathbf {x}}} \in {{{\varvec{\Psi }}}}_{\text {Soil}} , \end{aligned}$$

29 $$\begin{aligned} \partial _{t}(1-\phi _a-\phi _w)= & {} -\, \varepsilon {\varvec{\nabla }} \cdot [ (1-\phi _a-\phi _w) \partial _t{{\mathbf {u}}}_s ] , \quad {{\mathbf {x}}} \in {{{\varvec{\Psi }}}}_{\text {Soil}} , \end{aligned}$$

with the constitutive poroelastic mechanical law represented as:

30 $$\begin{aligned}&{\varvec{\nabla }} \cdot \bigg [ \left( {\varvec{\nabla }} {{\mathbf {u}}}_s \right) + \left( {\varvec{\nabla }} {{\mathbf {u}}}_s \right) ^T + {\overline{\nu }} {\varvec{\nabla }} \cdot {\mathbf {u}}_s {\mathfrak {T}}- \varepsilon ^{-1} \left( S_w{p}_w {\mathfrak {T}} - S_a{p}_a {\mathfrak {T}} \right) \bigg ] =0, \nonumber \\&\quad {{\mathbf {x}}} \in {{{\varvec{\Psi }}}}_{\text {Soil}} , \end{aligned}$$

where the force balances and relative movement of the air and water in the mixture domain are represented as:

31 $$\begin{aligned} \phi _a \left( {{\mathbf {v}}}_a - \partial _t{{\mathbf {u}}}_s \right)= & {} -\, {\overline{\kappa }}_a {\varvec{\nabla }} {p}_a, \quad {{\mathbf {x}}} \in {{{\varvec{\Psi }}}}_{\text {Soil}}. \end{aligned}$$

32 $$\begin{aligned} \phi _w \left( {{\mathbf {v}}}_w - \partial _t{{\mathbf {u}}}_s \right)= & {} -\, {\overline{\kappa }}_w {\varvec{\nabla }} {p}_w, \quad {{\mathbf {x}}} \in {{{\varvec{\Psi }}}}_{\text {Soil}}. \end{aligned}$$

The relationship between water and air is linked based on the Van Genuchten water retention relationship:

33 $$\begin{aligned} S_w =\bigg \{ \Big [ {\overline{G}} (p_a - p_w) \Big ]^{\frac{1}{1-m}} +1 \bigg \}^{-m}. \end{aligned}$$

The nutrients in the system follow the convection–diffusion equation:

34 $$\begin{aligned} (\phi _w + b)\partial _{{t}} {c} + {c}\partial _{{t}} \phi _w = {\varvec{\nabla }} \cdot \left( \phi _w {\varvec{\nabla }} {c} \right) - {\overline{g}} c , \quad {{\mathbf {x}}} \in {{{\varvec{\Psi }}}}_{\text {Soil}} , \end{aligned}$$

where we have no flux through the tuber surface:

35 $$\begin{aligned} {\hat{\mathbf {n}}} \cdot \left( \phi _w {\varvec{\nabla }} {c} \right) = 0, \quad {{\mathbf {x}}} \in {{{\varvec{\Gamma }}}}_j . \end{aligned}$$

The soil solid phase displacement is equal to the increase in the tuber radius:

36 $$\begin{aligned} \left( 2 {\hat{\mathbf {n}}} \otimes {\hat{\mathbf {n}}} -{\mathfrak {T}} \right) \cdot {\mathbf {u}}_s = {\hat{\mathbf {n}}} (r_j-\overline{r^*}), \quad {{\mathbf {x}}} \in {{{\varvec{\Gamma }}}}_j . \end{aligned}$$

Neither water nor air is assumed to flow through the tuber surface:

37 $$\begin{aligned} {\hat{\mathbf {n}}} \cdot \left( {\varvec{\nabla }} {p}_w \right)= & {} 0, \quad {{\mathbf {x}}} \in {{{\varvec{\Gamma }}}}_j , \end{aligned}$$

38 $$\begin{aligned} {\hat{\mathbf {n}}} \cdot \left( {\varvec{\nabla }} {p}_a \right)= & {} 0, \quad {{\mathbf {x}}} \in {{{\varvec{\Gamma }}}}_j . \end{aligned}$$

The water and air velocities normal to the tuber surface also follow the rate of the tuber growth:

39 $$\begin{aligned} \left( 2 {\hat{\mathbf {n}}} \otimes {\hat{\mathbf {n}}} - {\mathfrak {T}} \right) \cdot {{\mathbf {v}}}_w= & {} {\hat{\mathbf {n}}} \partial _{{t}} {r}_j, \quad {{\mathbf {x}}} \in {{{\varvec{\Gamma }}}}_j , \end{aligned}$$

40 $$\begin{aligned} \left( 2 {\hat{\mathbf {n}}} \otimes {\hat{\mathbf {n}}} - {\mathfrak {T}} \right) \cdot {{\mathbf {v}}}_a= & {} {\hat{\mathbf {n}}} \partial _{{t}} {r}_j, \quad {{\mathbf {x}}} \in {{{\varvec{\Gamma }}}}_j . \end{aligned}$$

Finally, the tuber growth rate is based on the rate of nutrient uptake out of the system:

41 $$\begin{aligned} \partial _{{t}} {r}_j = \frac{{\overline{\alpha }}}{4 \pi r_j^2} \int _{{{{\varvec{\Psi }}}}_{{\text {Soil}}_j}} {c} \ \mathrm{d}{{{\varvec{\Psi }}}}_{{\text {Soil}}_j}. \end{aligned}$$

Here, the system was non-dimensionalised as follows:

42 $$\begin{aligned} {\overline{\lambda _c}}&=\frac{\lambda _c G l_x^2}{D}, \, {\overline{p_r}} =\frac{p_r}{G}, \, {\overline{\nu }}=\frac{\nu }{1-2\nu }, \, {\overline{\kappa }}_a = \frac{\kappa _a G \varepsilon ^{-1}}{D \mu _a}, \, {\overline{\kappa }}_w = \frac{\kappa _w G \varepsilon ^{-1}}{D \mu _w},\nonumber \\ {\overline{G}}&=\frac{G}{p_c},\, {\overline{g}} = \frac{g l_x^2}{D}, {\overline{r^*}} = \frac{r^*}{l_y}, {\overline{\alpha }} = \frac{c_{\text {max}} \alpha g l_x^2}{D}. \end{aligned}$$

### Parameter Estimation

Here, we estimate the parameters contained in Eqs. (27)–(41) in order to determine the magnitude of influence each parameter has on the system of equations. Since this model is motivated by the growth of potato tubers in soil, we assess the parameter values for silt soils as potatoes are frequently grown in this soil type (Shock et al. 1998).

Potato plants are typically grown in ridge and furrow type systems and are contained in the plough layer of soil, which is the top 30 cm of soil (Lesczynski and Tanner 1976). Hence, we choose the macroscopic length scale to be $l_x \approx 0.3 \text { m}$. Similarly, we assume that the tubers have an inter-tuber distance that is substantially less than the total length of the plough layer. We choose an inter-tuber distance of approximately $l_y \approx 0.05 \text { m}$, resulting in the ratio of the two length scales to be $\varepsilon \approx 0.1$. We also assume an initial tuber radius of $r^* = {\mathcal {O}}(0.05) \text { m} < l_y$.

Values for the Poisson ratio of silt soils are approximately $0.3 \lesssim \nu \lesssim 0.35$ (Essien et al. 2014), and the shear modulus is $G \approx 1 \times 10^7 \ \text {Pa}$ (Vardanega and Bolton 2013). Furthermore, typical characteristic suction pressures for silt soils are approximately $p_c \approx 3 \times 10^4 \ \text {Pa}$ (Van Genuchten 1980), with soil permeabilities of $\kappa _w \approx \kappa _a \approx 5 \times 10^{-14} \ \text {m}^2$ (Van Genuchten 1980). The viscosity of water is $\mu _w \approx 10^{-3} \ \text {Pa} \ \text {s}$ and the viscosity of air is $\mu _a \approx 10^{-5} \ \text {Pa} \ \text {s}$.

One of the key nutrients responsible for plant growth and development is nitrogen (Nye and Tinker 1977). We choose to model this nutrient since plant growth is closely linked to abundance of nitrogen in soil. Nitrogen has a diffusion coefficient in soil water of $D \approx 2.5 \times 10^{-10} \ \text {m}^2 \ \text {s}^{-1}$ (Barber 1995). Furthermore, for the potato plant *Solanum tuberosum* L, the uptake rate of the nutrient nitrogen is $g \approx 1 \times 10^{-9} \ \text { s}^{-1}$ (Sattelmacher et al. 1990; Asfary et al. 1983). This was found to be in nitrogen concentrations in soil of $c_{\text {max}} \approx 10^{-1} \text {kg} \ \text {m}^{-3}$ (Asfary et al. 1983).

In early-stage growth of *Solanum tuberosum* L plants, the tuber radius growth rate is approximately $1 \times 10^{-9} \ \text {m} \ \text {s}^{-1}$ (Xu et al. 1998). If we assume that the quantity of nitrogen that is taken up by the plant is proportional to the growth of the tuber, then we can estimate the ratio between the rate of growth and the uptake, *i.e.*$\alpha \approx 1 \times 10^{1} \ \text {kg}^{-1} \ \text {m}^{-1}$ (Sattelmacher et al. 1990; Asfary et al. 1983).

Using the values above, we find that the parameters ${\overline{\kappa }}_a$ and ${\overline{\kappa }}_w$ in equations (31) and (32) are ${\overline{\kappa }}_a={\mathcal {O}}(10^9)$ and ${\overline{\kappa }}_w={\mathcal {O}}(10^7)$. This is significantly larger than the other terms in the equations. Hence, we rewrite Eqs. (31) and (32) so that,

43 $$\begin{aligned}&{\varvec{\nabla }} {p}_a \approx 0, \quad {{\mathbf {x}}} \in {{{\varvec{\Psi }}}}_{\text {Soil}}, \end{aligned}$$

44 $$\begin{aligned}&{\varvec{\nabla }} {p}_w \approx 0, \quad {{\mathbf {x}}} \in {{{\varvec{\Psi }}}}_{\text {Soil}}, \end{aligned}$$

which have the solutions $p_a=\text {constant}$ and $p_w=\text {constant}$, *i.e.* the consolidation of the soil is substantially faster than the diffusion of solutes. Since $p_w=\text {constant}$, we find that the sink term in Eq. (2) representing root uptake is constant, *i.e.*$\lambda _c({\tilde{p}}_w-p_r)=F$, where *F* is the water uptake rate by plant roots. The uptake rate of water from *Solanum tuberosum* L roots is $F \approx 1 \times 10^{-8} \ \text { s}^{-1}$ (Parker et al. 1989).

From Equation (33), the solutions $p_a=\text {constant}$ and $p_w=\text {constant}$ result in $S_w = \text {constant}$, and since $S_w + S_a = 1$, this leads to $S_a = \text {constant}$. Although $S_w$ is constant, $\phi _w$ will still change as a function of the changing domain geometry. Substituting 44 into 32 renders the domain water content to become dependent on the solid phase displacements:

45 $$\begin{aligned} \partial _t{{\mathbf {u}}}_s = {\mathbf {v}}_w, \quad {{\mathbf {x}}} \in {{{\varvec{\Psi }}}}_{\text {Soil}}. \end{aligned}$$

Thus, we reduce the system of Eqs. (27)–(41) to

46 $$\begin{aligned}&\partial _{t}\phi _w = - \varepsilon {\varvec{\nabla }} \cdot \left( \phi _w \partial _t{{\mathbf {u}}}_s \right) - {\overline{F}}, \quad {{\mathbf {x}}} \in {{{\varvec{\Psi }}}}_{\text {Soil}} , \end{aligned}$$

47 $$\begin{aligned}&{\varvec{\nabla }} \cdot \Big [ \left( {\varvec{\nabla }} {{\mathbf {u}}}_s \right) + \left( {\varvec{\nabla }} {{\mathbf {u}}}_s \right) ^T + {\overline{\nu }} {\varvec{\nabla }} \cdot {\mathbf {u}}_s {\mathfrak {T}} \Big ] =0, \quad {{\mathbf {x}}} \in {{{\varvec{\Psi }}}}_{\text {Soil}} , \end{aligned}$$

48 $$\begin{aligned}&(\phi _w + b)\partial _{{t}} {c} + {c}\partial _{{t}} \phi _w = {\varvec{\nabla }} \cdot \left( \phi _w {\varvec{\nabla }} {c} \right) - {\overline{g}} c , \quad {{\mathbf {x}}} \in {{{\varvec{\Psi }}}}_{\text {Soil}} , \end{aligned}$$

49 $$\begin{aligned}&\left( 2 {\hat{\mathbf {n}}} \otimes {\hat{\mathbf {n}}} -{\mathfrak {T}} \right) \cdot {\mathbf {u}}_s = {\hat{\mathbf {n}}} (r_j-\overline{r^*}), \quad {{\mathbf {x}}} \in {{{\varvec{\Gamma }}}}_j , \end{aligned}$$

50 $$\begin{aligned}&{\hat{\mathbf {n}}} \cdot \left( \phi _w {\varvec{\nabla }} {c} \right) = 0, \quad {{\mathbf {x}}} \in {{{\varvec{\Gamma }}}}_j , \end{aligned}$$

51 $$\begin{aligned}&\partial _{{t}} {r}_j = \frac{{\overline{\alpha }}}{4 \pi r_j^2} \int _{{{{\varvec{\Psi }}}}_{\text {Soil}_j}} {c} \ \mathrm{d}{{{\varvec{\Psi }}}}_{\text {Soil}_j}, \end{aligned}$$

where $${\overline{F}}=Fl_x^2/D$$.

Using the values discussed above, we find that the parameters contained in (46)–(51) have the approximate values

52 $$\begin{aligned}&{\overline{F}}={\mathcal {O}}(1), \ {\overline{\nu }}={\mathcal {O}}(1), \ {\overline{g}} = {\mathcal {O}}(1), \overline{r^*} = {\mathcal {O}}(1), {\overline{\alpha }} = {\mathcal {O}}(1). \end{aligned}$$

For the remainder of this study, Eqs.  (46)–(51) will be referred to as the ‘full set’ of equations to describe solute movement and tuber growth.

### Homogenisation

In this section, we use multiple scale homogenisation to develop a set of averaged macroscale equations that describe the movement of nutrients and tuber growth in soil. From Equation (46), we observe that $\phi _w$ is affected by two mechanisms: firstly by soil compression due to the growth of the tuber, *i.e.*$\varepsilon {\varvec{\nabla }} \cdot \left( \phi _w {{\mathbf {v}}}_s \right)$, and secondly by root water uptake, *i.e.*${\overline{F}}$. From the non-dimensionalisation, we observe that the maximum displacement is bounded such that ${\mathbf {u}}_s \ll {\overline{F}}$. This leads to the results $\partial _t {\mathbf {u}}_s \ll {\overline{F}}$. If we consider a scenario when the tubers are not taking up water, Eq. 45 suggests that the primary change in water content is based on $\varepsilon {\varvec{\nabla }} \cdot (\phi _w\partial _t {\mathbf {u}}_s)$. Since $\varepsilon {\varvec{\nabla }} \cdot (\phi _w\partial _t {\mathbf {u}}_s) \ll \partial _t {\mathbf {u}}_s$ and $\partial _t {\mathbf {u}}_s \ll {\overline{F}}$, then it follows that ${\varepsilon \varvec{\nabla }} \cdot (\phi _w\partial _t {\mathbf {u}}_s) \ll {\overline{F}}$. Therefore, we find that the root water uptake term dominates the change in water content. Hence, for the homogenisation procedure, we neglect the term regarding soil compression, and the system of equations we homogenise reduces to

53 $$\begin{aligned}&\partial _{t}\phi _w = - {\overline{F}} , \quad {{\mathbf {y}}} \in {{{\varvec{\Omega }}}}_{\text {Soil}} , \end{aligned}$$

54 $$\begin{aligned}&(\phi _w + b)\partial _{{t}} {c} + {c}\partial _{{t}} \phi _w = {\varvec{\nabla }} \cdot \left( \phi _w {\varvec{\nabla }} {c} \right) - {\overline{g}} c, \quad {{\mathbf {y}}} \in {{{\varvec{\Omega }}}}_{\text {Soil}} , \end{aligned}$$

55 $$\begin{aligned}&{\hat{\mathbf {n}}} \cdot \left( \phi _w {\varvec{\nabla }} {c} \right) = 0, \quad {{\mathbf {y}}} \in {{{\varvec{\Gamma }}}} , \end{aligned}$$

56 $$\begin{aligned}&\partial _{{t}} {r} = \frac{{\overline{\alpha }}}{4 \pi r^2} \int _{{{{\varvec{\Omega }}}}_{\text {Soil}}} {c} \ \mathrm{d}{{{\varvec{\Omega }}}}_{\text {Soil}}. \end{aligned}$$

57 $$\begin{aligned}&\text {periodic} \ {\mathbf {y}} \in \partial {{\varvec{\Omega }}}_E . \end{aligned}$$

To validate this assumption, we compare the full set of Eqs. (46)–(51) to the homogenised system of equations derived from (53)–(57) in the following section. We highlight that the horizontal boundaries for the full model preserve the periodicity presented here.

We observe there are two different length scales present in the geometry ${\tilde{{\varvec{\Psi }}}}$, the macroscale $l_x$ and the microscale $l_y$. Any change of ${\mathcal {O}}(1)$ on the length scale $l_x$ will result in a ${\mathcal {O}}(\varepsilon )$ change on the length scale $l_y$. We can formalise this by assuming that the dependent variables $\phi _w$, *c* and *r* are functions of a small scale ${\mathbf {y}}$ and a large-scale ${\mathbf {x}}$. We denote the unit cell ${{\varvec{\Omega }}}$ representing the microscale domain ${\mathbf {y}} \in {{\varvec{\Omega }}} \equiv [-1/2, 1/2]^3$. Using the two length scales and chain rule, the gradient operator is written as

58 $$\begin{aligned} {\varvec{\nabla }} = {\varvec{\nabla }}_{{\mathbf {x}}} + \varepsilon ^{-1}{\varvec{\nabla }}_{{\mathbf {y}}}. \end{aligned}$$

Furthermore, we expand $\phi _w$, *c* and *r* such that,

59 $$\begin{aligned} \phi _w= & {} \phi _{w_0} + {\mathcal {O}}(\varepsilon ), \end{aligned}$$

60 $$\begin{aligned} c= & {} c_0 + \varepsilon c_1 + \varepsilon ^2 c_2 + {\mathcal {O}}(\varepsilon ^3), \end{aligned}$$

61 $$\begin{aligned} r= & {} r_0 + {\mathcal {O}}(\varepsilon ). \end{aligned}$$

The first step of the homogenisation procedure is to determine the most dominant terms in the system of Eqs. (53)–(57). To do this, we substitute Eqs. (59)–(61) into (53)–(57), collecting the largest terms ${\mathcal {O}}(\varepsilon ^{-2})$. This results in the system of equations

62 $$\begin{aligned}&{\varvec{\nabla }}_{{\mathbf {y}}} \cdot (\phi _{w_0} {\varvec{\nabla }}_{{\mathbf {y}}} c_0)=0 , \quad {{\mathbf {y}}} \in {{{\varvec{\Omega }}}}_{\text {Soil}} , \end{aligned}$$

63 $$\begin{aligned}&{\hat{\mathbf {n}}} \cdot (\phi _{w_0} {\varvec{\nabla }}_{{\mathbf {y}}} c_0)=0, \quad {{\mathbf {y}}} \in {{{\varvec{\Gamma }}}} , \end{aligned}$$

64 $$\begin{aligned}&\text {periodic}\ {\mathbf {y}} \in \partial {{\varvec{\Omega }}}_E . \end{aligned}$$

*Theorem* Equations (62)–(64) have the solution $c_0 = c_0({\mathbf {x}},t)$, *i.e.*$c_0$ has large-scale dependence only.

#### Proof

We observe from (62) that

65 $$\begin{aligned} \int _{{{{\varvec{\Omega }}}}_{\text {Soil}}} c_0 {\varvec{\nabla }}_{{\mathbf {y}}} \cdot (\phi _{w_0} {\varvec{\nabla }}_{{\mathbf {y}}} c_0) \ \mathrm{d}{{{\varvec{\Omega }}}}_{\text {Soil}} =0 . \end{aligned}$$

Applying Green’s first identity to (65) yields

66 $$\begin{aligned}&\int _{\partial {{{\varvec{\Gamma }}}}} c_0 {\hat{\mathbf {n}}} \cdot (\phi _{w_0} {\varvec{\nabla }}_{{\mathbf {y}}} c_0) \ \mathrm{d}{{{\varvec{\Gamma }}}} + \int _{\partial {{{\varvec{\Omega }}}}_{E}} c_0 {\hat{\mathbf {n}}} \cdot (\phi _{w_0} {\varvec{\nabla }}_{{\mathbf {y}}} c_0) \ d\partial {{{\varvec{\Omega }}}}_{E} \nonumber \\&\quad - \int _{{{{\varvec{\Omega }}}}_{\text {Soil}}} {\varvec{\nabla }}_{{\mathbf {y}}} c_0 \cdot (\phi _{w_0} {\varvec{\nabla }}_{{\mathbf {y}}} c_0) \ \mathrm{d}{{{\varvec{\Omega }}}}_{\text {Soil}} =0 . \end{aligned}$$

Using (63) and (64), we find

67 $$\begin{aligned} \int _{{{{\varvec{\Omega }}}}_{\text {Soil}}} {\varvec{\nabla }}_{{\mathbf {y}}} c_0 \cdot (\phi _{w_0} {\varvec{\nabla }}_{{\mathbf {y}}} c_0) \ \mathrm{d}{{{\varvec{\Omega }}}}_{\text {Soil}} =0 . \end{aligned}$$

Equation (67) can be expressed as

68 $$\begin{aligned} \int _{{{{\varvec{\Omega }}}}_{\text {Soil}}} \phi _{w_0} ||{\varvec{\nabla }}_{{\mathbf {y}}} c_0 ||^2_2 \ \mathrm{d}{{{\varvec{\Omega }}}}_{\text {Soil}} =0 , \end{aligned}$$

where $||\cdot ||_2$ is the Euclidean norm, *i.e.*$||{\mathbf {x}} ||_2 = \sqrt{\langle {\mathbf {x}}, {\mathbf {x}} \rangle } = \sqrt{x^2_1 + ... + x^2_n}$. In order to satisfy (68), $||{\varvec{\nabla }}_{{\mathbf {y}}} c_0 ||^2_2 = 0$. By definition, $||{\mathbf {x}} ||_2 = 0 \iff {\mathbf {x}}=0$, hence

69 $$\begin{aligned} ||{\varvec{\nabla }}_{{\mathbf {y}}} c_0 ||^2_2 = 0. \quad \Rightarrow \quad {\varvec{\nabla }}_{{\mathbf {y}}} c_0 = 0. \end{aligned}$$

Therefore, $$c_0=c_0({\mathbf {x}}, t)$$. $$\square $$

From the theorem above, we observe that $c_0$ has large-scale dependence only and is independent of the small scale ${\mathbf {y}}$; however, we receive no other information regarding the solution of $c_0$.

To proceed with the homogenisation methodology, we collect the next most dominant terms in the system of equations. This is achieved by collecting terms ${\mathcal {O}}(\varepsilon ^{-1})$ and using the results ${\varvec{\nabla }}_{{\mathbf {y}}} c_0=0$, *i.e.*

70 $$\begin{aligned}&{\varvec{\nabla }}_{{\mathbf {y}}} \cdot ( \phi _{w_0}{\varvec{\nabla }}_{{\mathbf {y}}} c_1 + \phi _{w_0}{\varvec{\nabla }}_{{\mathbf {x}}} c_0)=0, \quad {{\mathbf {y}}} \in {{{\varvec{\Omega }}}}_{\text {Soil}} , \end{aligned}$$

71 $$\begin{aligned}&{\hat{\mathbf {n}}} \cdot (\phi _{w_0} {\varvec{\nabla }}_{{\mathbf {y}}} c_1 +\phi _{w_0} {\varvec{\nabla }}_{{\mathbf {x}}} c_0)=0, \quad {{\mathbf {y}}} \in {{{\varvec{\Gamma }}}} , \end{aligned}$$

72 $$\begin{aligned}&\text {periodic} \ {\mathbf {y}} \in \partial {{\varvec{\Omega }}}_E . \end{aligned}$$

To ensure (70)–(72) form a well-posed problem, *i.e.* the equations have a solution that agrees with the boundary conditions, we check the solvability of the system. We can show the system is well-posed by applying the divergence theorem to equation (70) and use the boundary condition (71) such that,

73 $$\begin{aligned}&\int _{{{{\varvec{\Omega }}}}_{\text {Soil}}}{\varvec{\nabla }}_{{\mathbf {y}}} \cdot ( \phi _{w_0}{\varvec{\nabla }}_{{\mathbf {y}}} c_1 + \phi _{w_0} {\varvec{\nabla }}_{{\mathbf {x}}} c_0) \ \mathrm{d}{{{\varvec{\Omega }}}}_{\text {Soil}} \nonumber \\&\quad = \int _{\partial {{{\varvec{\Omega }}}}_{\text {Soil}}} {\hat{\mathbf {n}}} \cdot (\phi _{w_0} {\varvec{\nabla }}_{{\mathbf {y}}} c_1 + \phi _{w_0} {\varvec{\nabla }}_{{\mathbf {x}}} c_0) \ d\partial {{{\varvec{\Omega }}}}_{\text {Soil}} =0. \end{aligned}$$

Next we choose to rescale $c_1$ such that,

74 $$\begin{aligned} c_1({\mathbf {x}}, {\mathbf {y}}) = \sum _{k=1}^3 \chi _k ({\mathbf {y}}) \partial _{x_{k}}c_0 + \bar{c_1}({\mathbf {x}}), \end{aligned}$$

where $\bar{c_1}({\mathbf {x}})$ is the large-scale component of $c_1({\mathbf {x}}, {\mathbf {y}})$. Substituting (74) into (70)–(72) yields the cell problem for $\chi _k$

75 $$\begin{aligned}&{\varvec{\nabla }}_{{\mathbf {y}}} \cdot ({\varvec{\nabla }}_{{\mathbf {y}}} {\chi _k} + {\hat{\mathbf {e}}}_k) =0, \quad {{\mathbf {y}}} \in {{{\varvec{\Omega }}}}_{\text {Soil}} , \end{aligned}$$

76 $$\begin{aligned}&{\hat{\mathbf {n}}} \cdot ({\varvec{\nabla }}_{{\mathbf {y}}} {\chi _k} + {\hat{\mathbf {e}}}_k ) =0, \quad {{\mathbf {y}}} \in {{{\varvec{\Gamma }}}} , \end{aligned}$$

77 $$\begin{aligned}&\text {periodic} \ {\mathbf {y}} \in \partial {{\varvec{\Omega }}}_E , \end{aligned}$$

where ${\hat{\mathbf {e}}}_k$ is the unit vector.

We note that the tubers grow in the soil domain; hence, the cell problem solution $\chi _k$ is dependent on the radius of the tuber. Since the cell problem is a representation of the impedance of nutrient movement due to tuber obstruction, and as the tuber grows, the impact on nutrient transport will change; therefore, we have the relationship $\chi _k=\chi _k(r)$, *i.e.* the cell problem solution is dependent on the radius of the tuber.

The last step of the homogenisation procedure is to collect terms ${\mathcal {O}}(\varepsilon ^0)$, *i.e.*

78 $$\begin{aligned}&\partial _{t}\phi _{w_0} = - {\overline{F}} , \quad {{\mathbf {y}}} \in {{{\varvec{\Omega }}}}_{\text {Soil}} , \end{aligned}$$

79 $$\begin{aligned}&(\phi _{w_0}+b)\partial _t c_0 + c_0 \partial _t \phi _{w_0} \nonumber \\&\quad = {\varvec{\nabla }}_{{\mathbf {y}}} \cdot (\phi _{w_0} {\varvec{\nabla }}_{{\mathbf {y}}} c_2 + \phi _{w_0} {\varvec{\nabla }}_{{\mathbf {x}}} c_1) \nonumber \\&\qquad +\, {\varvec{\nabla }}_{{\mathbf {x}}} \cdot (\phi _{w_0} {\varvec{\nabla }}_{{\mathbf {y}}} c_1 + \phi _{w_0} {\varvec{\nabla }}_{{\mathbf {x}}} c_0) - {\overline{g}} c, \quad {{\mathbf {y}}} \in {{{\varvec{\Omega }}}}_{\text {Soil}}, \end{aligned}$$

80 $$\begin{aligned}&{\hat{\mathbf {n}}} \cdot (\phi _{w_0} {\varvec{\nabla }}_{{\mathbf {y}}} c_2 + \phi _{w_0} {\varvec{\nabla }}_{{\mathbf {x}}} c_1)= 0, \quad {{\mathbf {y}}} \in {{{\varvec{\Gamma }}}} , \end{aligned}$$

81 $$\begin{aligned}&\text {periodic} \ {\mathbf {y}} \in \partial {{\varvec{\Omega }}}_E , \end{aligned}$$

82 $$\begin{aligned}&\partial _{{t}} {r_0} = \frac{{\overline{\alpha }}}{4 \pi r_0^2} \int _{{{{\varvec{\Omega }}}}_{S}} {c_0} \ \mathrm{d}{{{\varvec{\Omega }}}}_{S}. \end{aligned}$$

To check (78)–(82) provide a well-posed problem, we check the solvability of the system of equations. To do this, we apply the divergence theorem to (79)

83 $$\begin{aligned}&\int _{{{{\varvec{\Omega }}}}_{\text {Soil}}}(\phi _{w_0}+b)\partial _t c_0 + c_0 \partial _t \phi _{w_0} \ \mathrm{d}{{{\varvec{\Omega }}}}_{\text {Soil}} \nonumber \\&\quad = \int _{{{{\varvec{\Omega }}}}_{\text {Soil}}}{\varvec{\nabla }}_{{\mathbf {y}}} \cdot (\phi _{w_0} {\varvec{\nabla }}_{{\mathbf {y}}} c_2 + \phi _{w_0} {\varvec{\nabla }}_{{\mathbf {x}}} c_1) \ \mathrm{d}{{{\varvec{\Omega }}}}_{\text {Soil}} \nonumber \\&\qquad +\int _{{{{\varvec{\Omega }}}}_{\text {Soil}}} {\varvec{\nabla }}_{{\mathbf {x}}} \cdot (\phi _{w_0} {\varvec{\nabla }}_{{\mathbf {y}}} c_1 + \phi _{w_0} {\varvec{\nabla }}_{{\mathbf {x}}} c_0)\ \mathrm{d}{{{\varvec{\Omega }}}}_{\text {Soil}} - \int _{{{{\varvec{\Omega }}}}_{\text {Soil}}} {\overline{g}} c \ \mathrm{d}{{{\varvec{\Omega }}}}_{\text {Soil}} , \end{aligned}$$

and using boundary condition (80) yields

84 $$\begin{aligned}&\int _{{{{\varvec{\Omega }}}}_{\text {Soil}}}(\phi _{w_0}+b)\partial _t c_0 + c_0 \partial _t \phi _{w_0} \ \mathrm{d}{{{\varvec{\Omega }}}}_{\text {Soil}} \nonumber \\&\quad = \int _{{{{\varvec{\Omega }}}}_{\text {Soil}}}{\varvec{\nabla }}_{{\mathbf {x}}} \cdot (\phi _{w_0} {\varvec{\nabla }}_{{\mathbf {y}}} c_1 + \phi _{w_0} {\varvec{\nabla }}_{{\mathbf {x}}} c_0) \ \mathrm{d}{{{\varvec{\Omega }}}}_{\text {Soil}} \nonumber \\&\qquad - \int _{{{{\varvec{\Omega }}}}_{\text {Soil}}} {\overline{g}} c \ \mathrm{d}{{{\varvec{\Omega }}}}_{\text {Soil}}. \end{aligned}$$

We define

85 $$\begin{aligned} ||{{{\varvec{\Omega }}}}_{\text {Soil}} ||= ||{{{\varvec{\Omega }}}}_{\text {Soil}} (r) ||= \int _{{{{\varvec{\Omega }}}}_{\text {Soil}}} \ \mathrm{d}{{{\varvec{\Omega }}}}_{\text {Soil}}, \end{aligned}$$

to be the volume integral of the cell problem, which is dependent on the radius of the tuber. It follows that (84) can be written as

86 $$\begin{aligned}&||{{{\varvec{\Omega }}}}_{\text {Soil}} ||\big [ ( \phi _{w_0}+b)\partial _t c_0 + c_0 \partial _t \phi _{w_0} \big ] \nonumber \\&\quad = \frac{\partial }{\partial x_i} \int _{{{{\varvec{\Omega }}}}_{\text {Soil}}} \bigg [ \phi _{w_0} \left( \frac{\partial c_0}{\partial x_i} + \frac{\partial \chi _j}{\partial y_i} \frac{\partial c_0}{\partial x_j} \right) \bigg ] \ \mathrm{d}{{{\varvec{\Omega }}}}_{\text {Soil}} - ||{{{\varvec{\Omega }}}}_{\text {Soil}} ||{\overline{g}} c_0, \quad {{\mathbf {y}}} \in {{{\varvec{\Omega }}}}_{\text {Soil}} . \end{aligned}$$

This results in the approximate equations for $\phi _{w_0}$, $c_0$ and $r_0$

87 $$\begin{aligned}&\partial _{t}\phi _{w_0} = - {\overline{F}} , \quad {{\mathbf {y}}} \in {{{\varvec{\Omega }}}}_{\text {Soil}} , \end{aligned}$$

88 $$\begin{aligned}&||{{{\varvec{\Omega }}}}_{\text {Soil}} (r_0) ||\big [ ( \phi _{w_0}+b)\partial _t c_0 + c_0 \partial _t \phi _{w_0} \big ] \nonumber \\&\quad = \phi _{w_0}{\varvec{\nabla }}_{{\mathbf {x}}} \cdot \left( {\mathfrak {D}}_{\mathfrak {e}}(r_0) {\varvec{\nabla }}_{{\mathbf {x}}} c_0 \right) \nonumber \\&\qquad -\, ||{{{\varvec{\Omega }}}}_{\text {Soil}} (r_0) ||{\overline{g}} c_0, \quad {{\mathbf {y}}} \in {{{\varvec{\Omega }}}}_{\text {Soil}} , \end{aligned}$$

89 $$\begin{aligned}&\partial _{{t}} {r_0} = \frac{{\overline{\alpha }}}{4 \pi r_0^2} ||{{{\varvec{\Omega }}}}_{\text {Soil}} (r_0) ||{c_0}, \end{aligned}$$

where

90 $$\begin{aligned} {\mathfrak {D}}_{\mathfrak {e}}(r_0) = \int _{{{{\varvec{\Omega }}}}_{\text {Soil}}} {\mathfrak {T}} +{\varvec{\nabla }}_{{\mathbf {y}}} {\chi _k}(r_0) \otimes {\hat{\mathbf {e}}}_k \ \mathrm{d}{{{\varvec{\Omega }}}}_{\text {Soil}}, \end{aligned}$$

for $$k=(1,\ldots ,3)$$.

Here, the averaged terms $||{{{\varvec{\Omega }}}}_{\text {Soil}} (r_0) ||$ and ${\mathfrak {D}}_{\mathfrak {e}}(r_0)$ are parameterised from the cell problem (75)–(77). This result identifies that Eqs. (78)–(82) provide a well-posed problem if and only if the system of Eqs. (87)–(90) has a solution. For the remainder of this study, Eqs. (87)–(90) will be referred to as the ‘homogenised set’ of equations to describe solute movement and tuber growth.

**Fig. 3:**
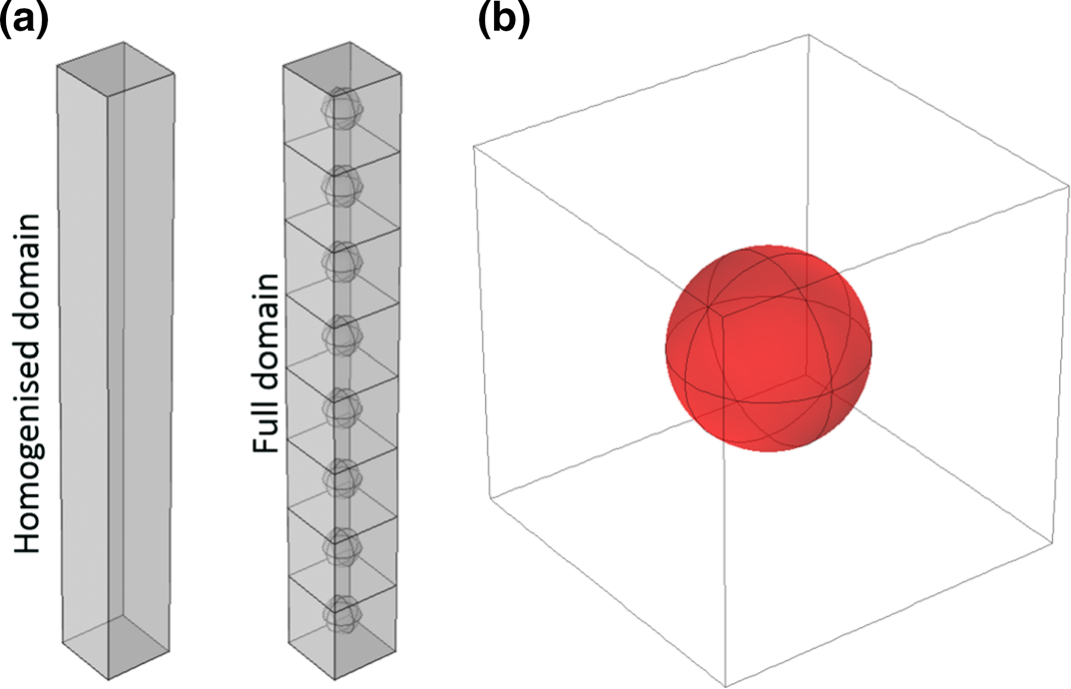
The geometries used to validate the homogenisation procedure **a** the approximate Eqs. (87)–(90) are solved on the left geometry, whereas the original set of Eqs. (46)–(51) are solved on the right geometry that contains potato tubers. **b** The cell problem is solved on a single unit cell that contains a potato tuber (coloured in red). Comparisons between the homogenised model and the full model were done by analysing the concentrations along the of the domain running down the vertical axes (Color figure online)

## Validation of the Homogenisation Procedure

We validate the mathematical steps used in the homogenisation procedure by comparing the homogenised set of Eqs. (87)–(90) to the full set of Eqs. (46)–(51). We consider multiple comparisons by varying parameters for the buffer power *b*, root uptake rate ${\overline{F}}$ and initial volumetric water content $\phi _{w}|_{t=0}$ to examine the accuracy of the averaging procedure.

We generate two geometries, one for the full set of Eqs. (46)–(51) containing potato tubers and the other uniform geometry for the homogenised Eqs. (87)–(90). We choose the domain length of each geometry to be composed of eight periodic cells. Due to the homogenisation procedure, the approximate Eqs. (87)–(90) do not require any tubers as the influence of the microscale geometry is contained in the parameterised terms $||{{{\varvec{\Omega }}}}_{\text {Soil}} (r_0) ||$ and ${\mathfrak {D}}_{\mathfrak {e}}(r_0)$. Shown in Fig. 3 are the geometries used to validate the homogenisation procedure.

Lastly, we numerically illustrate that the full solution tends towards the homogenised solution as $\varepsilon \rightarrow 0$. Since $\varepsilon = \frac{l_y}{l_x}$, it suffices to show that the solutions become closer as $l_x \rightarrow \infty$, as this implies that $\varepsilon \rightarrow 0$. We begin with by setting $l_x = 0.3$, where the domain of the full solution can only consist of 3 tubers. We incrementally increase the domain size up to $l_x = 0.8$, where the domain consists of 8 tubers. We take the percent difference between the homogenised solution for the concentration profile as:

91 $$\begin{aligned} d_p= \frac{\Vert c^{(\varepsilon )}-c^{(\varepsilon )}_0 \Vert _\infty }{\Vert c^{(\varepsilon )} \Vert _\infty }\times 100 \% , \quad {{\mathbf {x}}} \in {{{\varvec{\Omega }}}}_{\text {Soil}} , \end{aligned}$$

where $c^{(\varepsilon )}$ is the solute concentration profile in the full solution as a function of a given $\epsilon$, $c^{(\varepsilon )}_0$ is the solute concentration profile based on the homogenised solution, $\Vert c^{(\varepsilon )}-c^{(\varepsilon )}_0 \Vert _\infty$is the largest difference between the two solutions for all time points in the whole domain for a fixed $\varepsilon$ value, and $\Vert c^{(\varepsilon )} \Vert _\infty$ is the supremum concentration value for all time in the full domain for the full solution for a fixed $\varepsilon$ value.

To solve the systems of equations, we use the finite element package COMSOL Multiphysics^®^ 5.3 (www.comsol.com). We run our full model with a mesh consisting of 21729 tetrahedral elements and 1405 for the homogenised model. Simulations were run using the MUMPS (Multifrontal Massively Parallel Sparse) direct solver for a fully coupled physical system. In this section, we describe the implementation of each set of equations and show a comparison between them.

### Full Equations

Implementation of the full set of Eqs. (46)–(51) requires the implementation of a complex moving boundary problem. This accounts for the uptake of nutrients by each tuber ${{\varvec{\Psi }}}_{p_j}$, the subsequent growth of ${{\varvec{\Psi }}}_{p_j}$ and the reduction in volumetric water content $\phi _w$. The geometry we impose the full set of equations on can be seen in Fig. 3a. However, we require two versions of this geometry, an undeformed geometry that is constant in time, and a deforming geometry that is dependent on tuber growth, since different components of the system (46)–(51) are solved on either an undeformed or deforming frame of reference. There are three main components that are required to be implemented in order to solve (46)–(51); these are the poroelastic equations, the compaction and deformation of soil, and the nutrient movement equations.

To implement the poroelastic equations (46)–(47) and (49) for the local displacement ${\mathbf {u}}^s$ and reduction in $\phi _w$ is straightforward, since these equations are solved on the undeformed geometry regardless of tuber size. Using this solution at each time step, we can prescribe a deformation (for the deforming geometry) within the soil domain to correspond with the increase in tuber size.

The nutrient equations (48) and (50)–(51) are solved on the deforming geometry to correspond with the growth of the tubers. However, these equations use the poroelastic solution from the undeformed geometry. Hence, we implement a reference frame change such that poroelastic solution can be mapped from the undeformed geometry to the deformed geometry. This allows us to solve the nutrient equations on the deformed geometry corresponding with the prescribed tuber deformation.

Since the nutrient equations are solved on a deforming geometry, we are required to ensure that *c* is conserved. This is achieved by making two alterations to (48) and (50). Firstly, we note Reynolds transport theorem

92 $$\begin{aligned} \frac{\mathrm {d}}{\mathrm {d}t} \int _{\theta (t)} {\mathbf {F}} \ \mathrm {d}V = \int _{\theta (t)} \frac{\partial {\mathbf {F}}}{\partial t} \ \mathrm {d}V + \int _{\partial \theta (t)} (\varvec{\omega } \cdot {\hat{\mathbf {n}}} ) {\mathbf {F}} \ \mathrm {d}A, \end{aligned}$$

where d*V* and d*A* are volume and surface elements, respectively, $\varvec{\omega }$ is the velocity of the surface element, ${\hat{\mathbf {n}}}$ is the normal vector pointing out of the geometry, ${\mathbf {F}}$ is any function of ${\mathbf {x}}$ and *t* and $\theta (t)$ is the domain. Reynolds transport theorem states that the change in nutrient concentration in a domain is equal to the change in concentration within the domain plus the rate at which nutrient is entering the domain. Applying equation (92) to the full set of equations we have leads to

93 $$\begin{aligned} \frac{\mathrm {d}}{\mathrm {d}t} \int _{{{\varvec{\Psi }}}_{\text {Soil}(t)}} c \ \mathrm{d}{{\varvec{\Psi }}}_{\text {Soil}(t)}= & {} \int _{{{\varvec{\Psi }}}_{\text {Soil}(t)}} \frac{\partial c}{\partial t} \ \mathrm{d}{{\varvec{\Psi }}}_{\text {Soil}(t)} \nonumber \\&+ \int _{\partial {{\varvec{\Psi }}}_{\text {Soil}(t)}} (\varvec{\omega }_{\text {mesh}} \cdot {\hat{\mathbf {n}}} ) c \ \mathrm{d} { \partial {{\varvec{\Psi }}}_{\text {Soil}(t)}}, \end{aligned}$$

where $\varvec{\omega }_{\text {mesh}}$ is the velocity of the boundaries ${{{\varvec{\Psi }}}}_{p_j}$. This requires us to adapt equation (50) so that,

94 $$\begin{aligned} {\hat{\mathbf {n}}} \cdot \left( \phi _w {\varvec{\nabla }} {c} \right) = - (\varvec{\omega }_{\text {mesh}} \cdot {\hat{\mathbf {n}}} ) c , \quad {{\mathbf {x}}} \in {{{\varvec{\Gamma }}}}_j . \end{aligned}$$

Equation (94) then satisfies the conservation law for moving boundaries.

Secondly, as ${{\varvec{\Psi }}}_{p_j}$ grows and ${{\varvec{\Psi }}}_S$ is deformed, this causes an advective movement effect on *c* within ${{\varvec{\Psi }}}_S$. This can be interpreted as the boundaries of the tubers and ${{\varvec{\Gamma }}}_j$ physically pushing the nutrients. Hence, we are required to add a conservative advection term to Eq. (48) accounting for the individual elements within the mesh moving, *i.e.*

95 $$\begin{aligned} (\phi _w + b)\partial _{{t}} {c} + {c}\partial _{{t}} \phi _w = {\varvec{\nabla }} \cdot \left( \phi _w {\varvec{\nabla }} {c} - \varvec{\omega }_{\text {mesh}} c \right) - {\overline{g}} c , \quad {{\mathbf {x}}} \in {{{\varvec{\Psi }}}}_{\text {Soil}}. \end{aligned}$$

This modified system of equations can then be successfully implemented to model coupled nutrient movement and poroelastic deformation from growing tubers.

### Homogenised Equations

The geometry used to simulate the homogenised set of equations can be seen in Fig. 3a. However, to solve the set of homogenised Eqs. (87)–(90), we are required to solve a series of cell problems, *i.e.* Eqs. (75)–(77), to calculate the terms $||{{{\varvec{\Omega }}}}_{\text {Soil}} (r_0) ||$ and ${\mathfrak {D}}_{\mathfrak {e}}(r_0)$ that parameterise Eqs. (88) and (89). Since the geometric properties of the domain ${{\varvec{\Omega }}}$ are contained in $||{{{\varvec{\Omega }}}}_{\text {Soil}} (r_0) ||$ and ${\mathfrak {D}}_{\mathfrak {e}}(r_0)$, we solve the cell problem for a series of different tuber radii to correspond with different levels of growth/displacement from the original tuber size. Using the results from the cell problems, we can construct interpolated functions to describe $||{{{\varvec{\Omega }}}}_{\text {Soil}} (r_0) ||$ and ${\mathfrak {D}}_{\mathfrak {e}}(r_0)$ as functions of the homogenised radius $r_0$.

### Results

To validate the homogenisation procedure, we compare the homogenised Eqs. (87)–(90) against the original set of Eqs. (46)–(51). We choose to run a series of case studies by varying the parameters $b, \ {\overline{F}}$ and $\phi _w|_{t=0}$. For the buffer power *b*, we choose the values $b\in \{0.5, 5 \}$ since this covers a range of buffer powers for the nutrients nitrogen, boron, magnesium, zinc and molybdenum (Barber 1995). From the non-dimensionalisation and parameter estimation, we observe the value for root water uptake is ${\overline{F}}={\mathcal {O}}(1)$. However, to test the homogenisation procedure, we select the values ${\overline{F}}\in \{0.1, 10 \}$ for low and high levels of water uptake, respectively. Finally, for the initial water content $\phi _w|_{t=0}$ we assign the values $\phi _w|_{t=0} \in \{0.4, 0.6 \}$ as these are approximate upper and lower bounds for silty soils (Das 2013).

In each of the simulations we impose a Dirichlet condition of $c=c_0=1$ on the top of each of the geometries shown in Fig. 3a. Additionally, we choose the initial non-dimensionalised tuber radius to be $\overline{r^*}=0.025$ and choose the remaining parameters to be ${\overline{g}}={\overline{\alpha }}=1$. We also impose a stop condition on each of the simulations so that when the non-dimensionalised volume of a tuber has doubled in magnitude, the simulation is terminated. Finally, in order to construct interpolated functions to describe $||{{{\varvec{\Omega }}}}_{\text {Soil}} (r_0) ||$ and ${\mathfrak {D}}_{\mathfrak {e}}(r_0)$ in Eqs. (88) and (89), we solve a series of 6 cell problems with varying sphere radii.

**Fig. 4:**
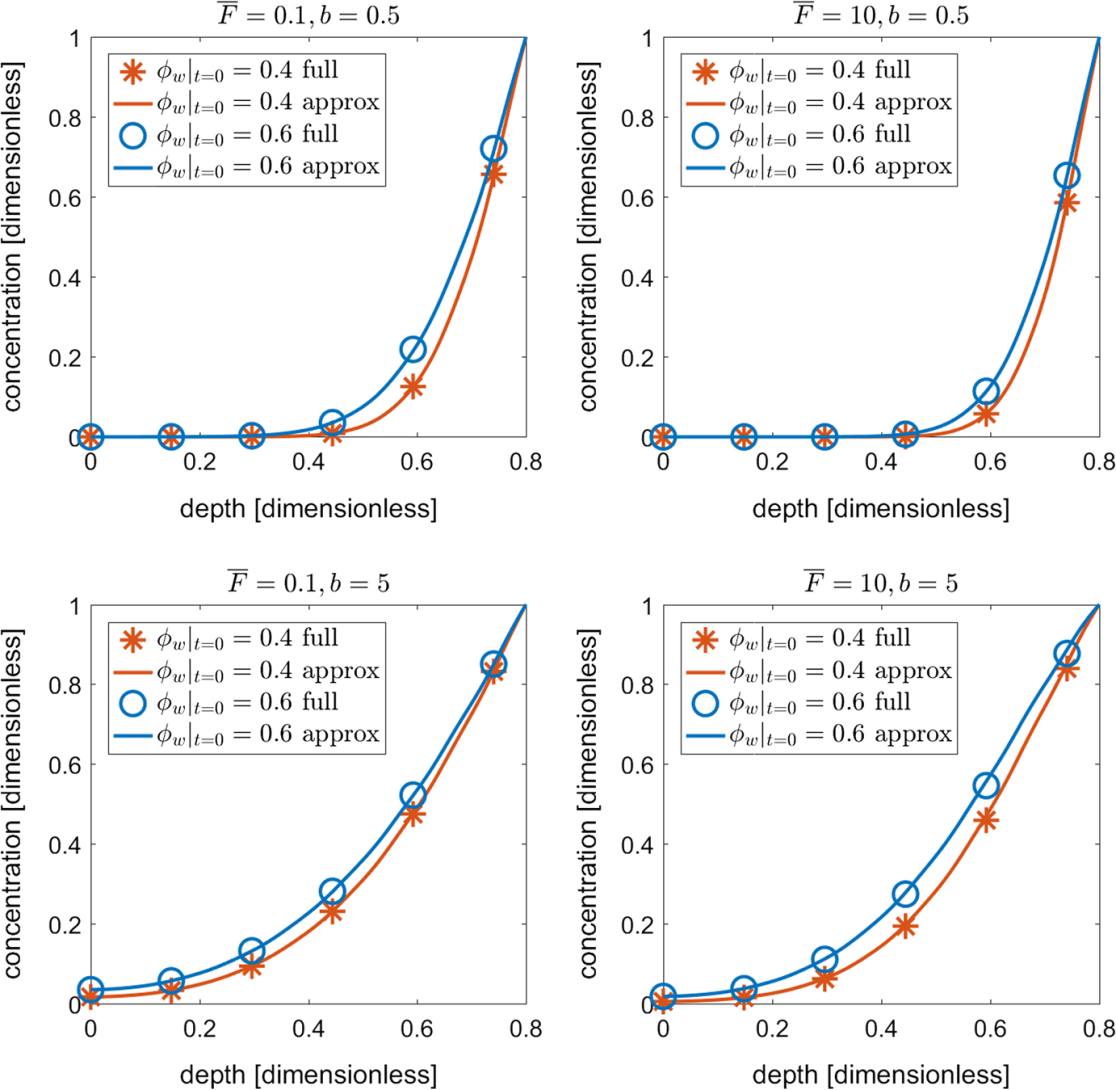
Validation of homogenised Eqs. (87)–(90) against the original set of Eqs. (46)–(51). The plots show the nutrient profile *c* and $c_0$ from the base to the top of the domains shown in Fig. 3 for a series of case studies using the parameter values $b\in \{0.5, 5\}$, ${\overline{F}}\in \{0.1, 10 \}$, $\phi _w|_{t=0} \in \{0.4, 0.6 \}$ (Color figure online)

Shown in Fig. 4 are the nutrient profiles for *c* and $c_0$ down the length of the geometries shown in Fig. 3a. We observe for all buffer powers, root uptake values and initial porosities, that the homogenised nutrient profile for $c_0$ is qualitatively identical to the full nutrient concentration *c*. We find there to be a maximum error of $\lesssim 2\%$ between the solutions across all scenarios.

**Fig. 5:**
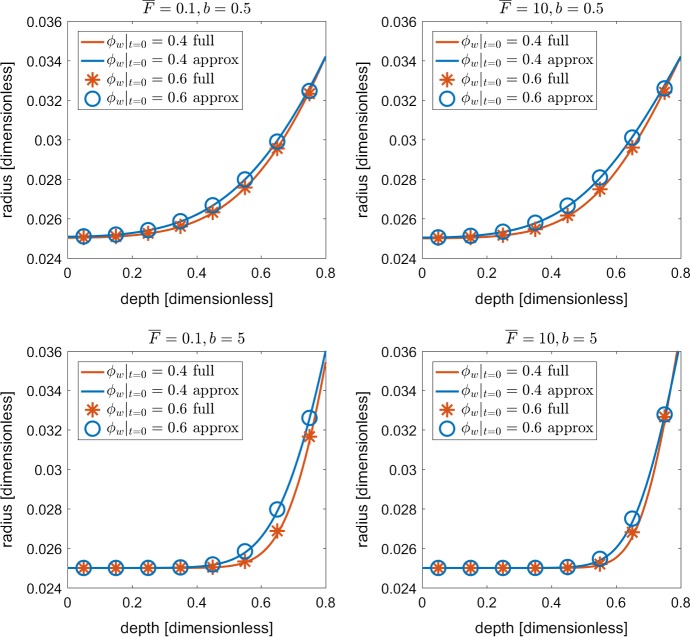
Validation of homogenised Eqs. (87)–(90) against the original set of Eqs. (46)–(51). The plots show the effective radius $r_0$ against the actual radius $r_j$ of the tubers from the base to the top of the domains shown in Fig. 3 for a series of case studies using the parameter values $b\in \{0.5, 5\}$, ${\overline{F}}\in \{0.1, 10 \}$, $\phi _w|_{t=0} \in \{0.4, 0.6 \}$ (Color figure online)

Additionally, shown in Fig. 5 are the individual tuber radii $r_j$ for the full set of equations against the effect radius $r_0$ from the homogenised equations. Similar to the results from Fig. 4, we find that the effective radius $r_0$ successfully captures the growth of each tuber within the full domain shown in Fig. 3a. We find there to be a maximum error of $\lesssim 2\%$ between the actual and effective tuber radius.

**Fig. 6:**
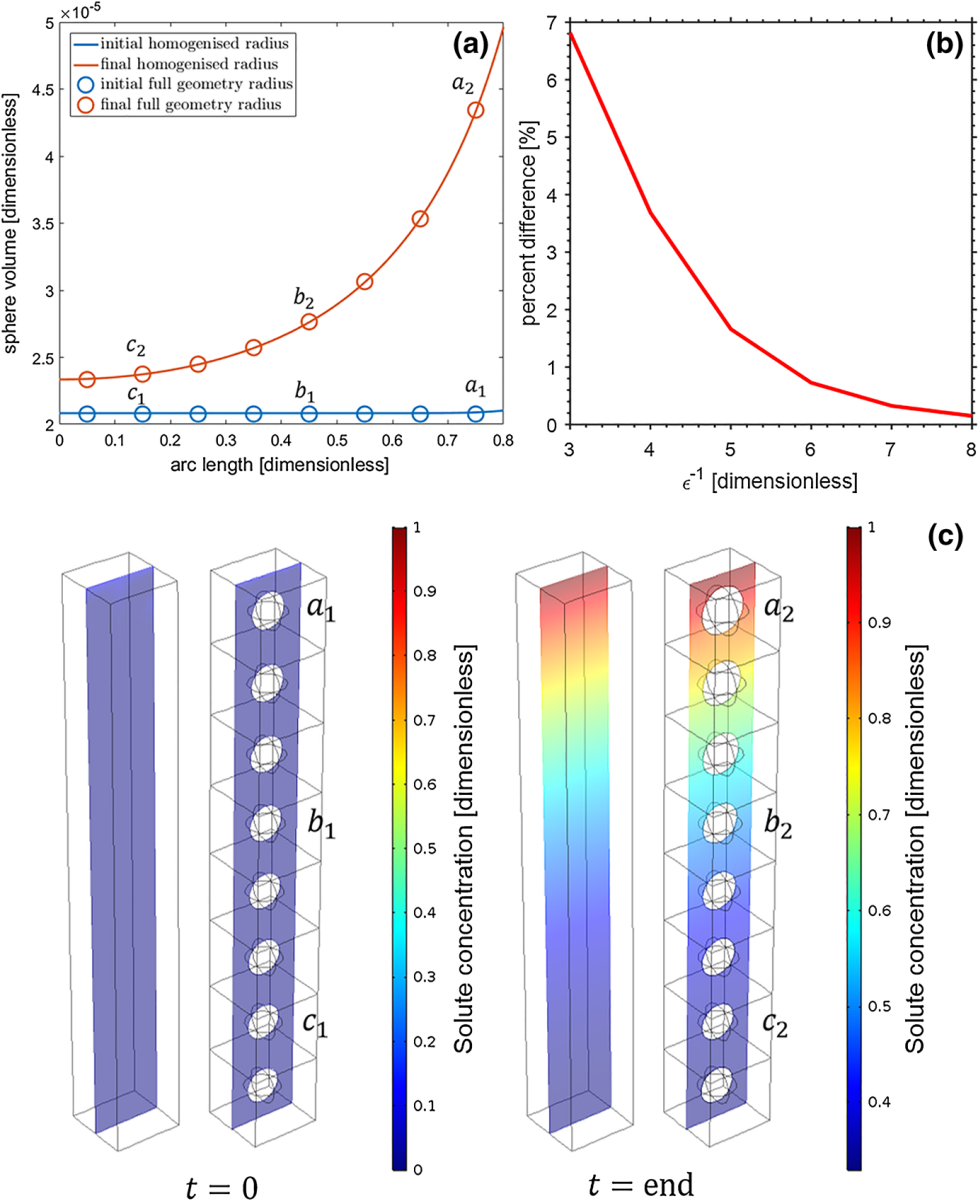
**a** Shown are the results for the actual and effective tuber volumes for the simulation using the parameters ${\overline{F}}=0.1, b=0.5$ and $\phi _w|_{t=0}=0.4$ at the beginning and end of the simulation. **b** The convergence of the full solution to the homogenised solution (presented as percent difference) as $\varepsilon \rightarrow 0$. **c** Shown are the results for the actual and effective solute concentration for the same simulation as (**a**). Additionally the geometries capturing the tuber growth are shown (Color figure online)

To highlight the accuracy of the homogenised set of equations, shown in Fig. 6 are detailed results for the simulation using the parameters ${\overline{F}}=0.1$, $b=0.5$ and $\phi _w|_{t=0}=0.4$. From Fig. 6a we observe that the effective radius $r_0$ is able to mimic the growth of the tubers in the full geometry. Figure 6b illustrates that as we have a system with more tubers, the full model converges to the homogenised model. For the 8 tuber scenario, the growing tubers can be seen in Fig. 6c, in which the tubers at the top of the full equation domain at the time point $t=\text {end}$ have grown substantially larger than those at the base of the domain. Furthermore, we find that the solute concentration profiles exhibit identical trains between the full and homogenised domains.

As a final confirmation of our model accuracy, we increased our mesh density from 21,729 tetrahedral elements to 49,218 elements in order to insure that our results are sufficiently accurate. Percent difference between the refined vs course solutions was on the order of 0.1%. This grants us confidence that our mesh is sufficiently resolved given the current problem. It is worth noting that our current simulations do not consider any automatic mesh refinement, as the deformations modelled do not result in any large aspect ratios. Future considerations could include more general geometries or greater deformations (Dehghani et al. 2018). This would be more significant when considering large deformations, which are more common in soil materials (Yu 2000).

**Fig. 7:**
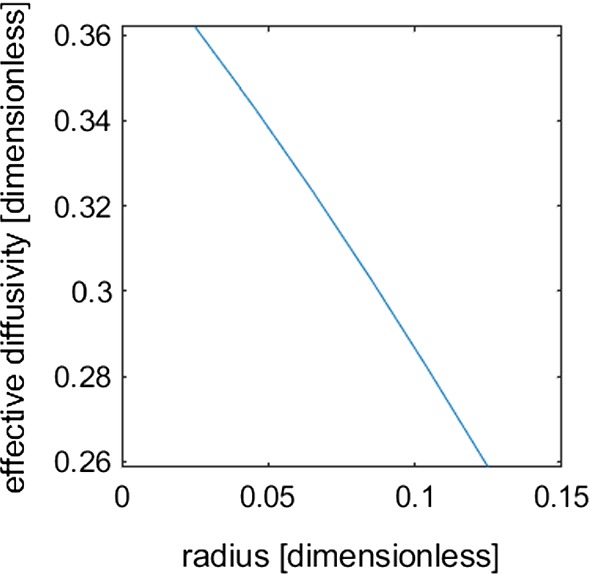
Effective homogenised diffusivity coefficient as a function of potato radius (Color figure online)

From Figs. 4 and 5, we observe that the homogenised equations successfully capture the nutrient movement and tuber growth in soil. However, the computation time between the two systems of equations differs by several orders of magnitude. We find that the full set of equations in three dimensions requires $\approx 5$ min (300 s) to solve one simulation for eight periodic cells. Conversely, solving the homogenised equations requires $\approx 10$ s to solve an analogous 3D simulation. Furthermore, the homogenised set of equations can be reduced to a 1D problem which will achieve the same results as the 3D problem due to the homogenisation procedure. We find that the computation time to solve the 1D problem is $\ll 1$ s, which is substantially faster than the full set of equations. However, a set of 3D cell problems is required to parameterise the homogenised set of equations for the terms $||{{{\varvec{\Omega }}}}_{\text {Soil}} ||$ and ${\mathfrak {D}}_{\mathfrak {e}}$. In this case study, we chose to conduct six cell problems for varying sphere radii. Each of the cell problems requires $\approx 10$  s to solve. However, these cell problems are only required to be solved once for each set of parameters. Hence, we find that the homogenised sets of equations can reduce the computation time substantially while retaining a high level of accuracy. Furthermore, we can highlight the influence that the tubers’ radii have on the effective homogenised diffusion coefficient (Fig. 7). Under more dramatic growth scenarios where tubers increase their radii by a factor of 5, the effective diffusion in the system could be reduced by as much as 30%.

## Discussion

In this study, we developed a physical model for potato tuber growth that couples water and nutrient uptake with mechanical growth of potatoes in soil. The explicit consideration of the potato growth in the soil domain creates a physical impedance to nutrient transport through the soil. The geometry and the surface sinks due to the presence of potatoes impede the effective transport of nutrients through the soil domain (Fig. 6). If impedance to diffusion caused by the potato tubers was not considered, we would incur an error between 175 and $300\%$ in the effective diffusion of the solute (where the effective diffusion is $D_\mathrm{eff}=\frac{\phi D}{\phi +b}$ in the case with no potatoes and $D_\mathrm{eff}^h = \frac{\phi (r_0){\mathfrak {D}}_{\mathfrak {e}}(r_0)}{(\phi (r_0)+b)||{{{\varvec{\Omega }}}}_{\text {Soil}(r_0)}||}$ when impedance to diffusion caused by growing tubers is homogenised). Furthermore, an error of up to $62.5\%$ in effective diffusion could occur if the tubers were modelled as a sphere with constant half of the final time-dependent radius. These errors in effective diffusion would greatly impact the predicted solute leaching or plant solute uptake of models, ignoring geometric impedance to diffusion.

One primary novelty associated with our growth model largely pertains to the growth domain locally external to our growing tubers. Similar studies have invoked a fluid–solid mechanical coupling to describe biological tissue as a porous medium, where cells are grow in an interstitial fluid (O’Dea et al. 2015; Penta et al. 2014). These models deal with a saturated fluid domain interacting with a solid cell that is able to grow based on either nutrient uptake (O’Dea et al. 2015) or a prescribed growth rate (Penta et al. 2014). Our model is applied to a partially saturated domain. Similar to O’Dea et al. (2015), our biological agents grow proportional to the rate of nutrient uptake. However, our potato tubers also take up water, which impacts the advective fluxes associated with the nutrient transport in the unsaturated soil domain. As the focus of our system is to obtain a geometrically simplified model through our homogenisation procedure, the final equations that arise are convection–diffusion equations. By choosing a different re-scaling approach, it may be possible to obtain a similar Darcy type expression as demonstrated by O’Dea et al. (2015) and Penta et al. (2014); however, this was not within the scope of this study.

Previous studies have coupled fluid and solid mechanical systems to infer not only the impact that a solid inclusion would have on the fluid flow, but also the mechanical deformations that fluid flow would induce on the solid inclusion (Royer et al. 2019; Chen et al. 2019). Authors have found that the homogenised system parameters are impacted by the distribution of inclusions in the domain. While our modelling scheme does not explicitly account for the mechanical response of the inclusions to externally applied stresses, the distribution of our potato tubers impacts the flow and transport coefficients in a similar manner as demonstrated in previous studies (Royer et al. 2019). It is worth noting that the growth behaviour of our modelled tubers implies a compensation for external stresses. Plant roots are known to respond to mechanical stresses by increasing their radii and reducing their length (Abdalla et al. 1969). This behaviour does not readily lend itself to a simple coupling between mechanical stresses and growth responses, and future work should be conducted to better quantify these contrasting effects.

The full system of equations in this paper required the implementation of a complex moving boundary problem. This required the use of multiple domains to solve different components of the equations, and subsequent mappings of solutions across domains. Not only does this system require considerable computational power to solve, the time required to correctly implement this system is substantial. This is due to ensuring conservation of mass and consistent mappings of solutions across domains. Using mathematical homogenisation, many of the more cumbersome modelling aspects were simplified into an effective media, where the tuber surfaces are treated as domain sinks, and the tuber geometries are accounted for in the diffusivity term shown in Figs. 4 and 5. Applying a similar method to a root system would facilitate a more rigorous quantification of bulk scale rhizosphere transport dynamics for both water and nutrients, generating better tools to disentangle the plant influence (rhizosphere soil) from the soil physical properties (bulk soil) (Koebernick et al. 2017).

Although the explicit model couples the poroelastic mechanical model to the transport equations for water and nutrients, a more specific mechanical coupling might be more appropriate to define the tubers expanding in partially saturated soil. Partially saturated soils are not subject to consolidation (Yu 2000); thus, considering the soil as an elasto-viscoplastic media may be important in this situation (Ghezzehei and Or 2000). Furthermore, the mechanical stresses likely exceed typical yield stress values found in soil under field saturation conditions (Ghezzehei and Or 2001). Previous models have utilised strictly linear-elastic parameters to quantify the mechanics of cavity expansion in unsaturated soil (Aravena et al. 2014); however, future work should attempt to remedy this by considering soil plasticity.

This study was motivated by the growth of tubers in soil; however, the system of equations is not limited to this particular problem. Other biological processes could also be modelled, including, but not limited too, clusters of lymph nodes swelling under an inflammatory response from a disease or virus moving through a biological tissue (Yang et al. 2014), the growth of roots in response to water and nutrients (Aravena et al. 2014; Drew and Saker 1978), or to model the effect of tumour growth on nutrient flow during angiogenesis (Alarcón et al. 2003).

Technical analysis regarding the homogenisation procedure showed encouraging results. Comparing the results from the homogenised sets of equations to the full set yielded less than about a 2% difference between nutrient concentrations at different depths, as shown in Fig. 4. Despite similarities in the results, the homogenised set of equations could be solved 1000 times faster than the full set of equations. Furthermore, the homogenised model could be physically scaled up with minimal increases to computational time, while increasing the domain size for the explicit geometry will substantially increase the computational time. This is important if we were to do combinatorial simulations spanning large numbers of soil and climate parameters to predict how potato crops grow. Thus, the averaged model will computationally allow extensive explorations of soil management and crop breeding strategies to be investigated *in silico*.
